# Hemodynamic Insights Into the Asymmetrical Medullary Vein Sign on T2* Imaging in Hyperacute M1 Occlusion

**DOI:** 10.7759/cureus.75775

**Published:** 2024-12-15

**Authors:** Keisuke Kadooka, Yoshito Arakaki, Yoichi Kikuchi, Takafumi Mitsutake, Michihiro Tanaka, Tatsuya Tanaka, Fumitaka Yamane, Akira Matsuno

**Affiliations:** 1 Department of Neuroendovascular Surgery, Kameda Medical Center, Kamogawa, JPN; 2 Department of Neurosurgery, International University of Health and Welfare Graduate School, Narita, JPN; 3 Department of Neurology, National Cerebral and Cardiovascular Center, Suita, JPN; 4 Department of Radiology, Kameda Medical Center, Kamogawa, JPN; 5 Department of Neurosurgery, Kameda Medical Center, Kamogawa, JPN; 6 Department of Neurosurgery, International University of Health and Welfare Narita Hospital, Narita, JPN; 7 Department of Neurosurgery, International University of Health and Welfare Atami Hospital, Atami, JPN

**Keywords:** acute ischemic stroke, anastomotic medullary vein, asymmetrical vein sign, mechanical thrombectomy, medullary vein, transcerebral vein

## Abstract

Background

In treating acute ischemic stroke (AIS), asymmetrical vein signs (AVS) on blood-oxygen-level-dependent imaging reflect increased deoxyhemoglobin levels due to increased oxygen extraction fraction. Meanwhile, although veins connecting pial and deep venous systems, such as transcerebral veins, are well studied, dynamic observation of these veins remains challenging. This study aimed to elucidate the venous flow of the deep white matter (DWM), focusing on medullary AVS in patients with hyperacute cardioembolic M1 occlusion.

Methods

This retrospective cross-sectional study involved 50 patients with AIS caused by M1 occlusion who received mechanical thrombectomy at Kameda Medical Center from July 2018 to December 2021. The study investigated medullary AVS and their association with angiographic collateral flow grades and occlusion locations. Welch's t-test was used for continuous variables, while Fisher's exact test was employed for categorical variables.

Results

A total of 41 patients were eligible for analysis. No significant association was found between medullary AVS and angiographic collateral flow grade (p=1.000); however, a significant association was observed between proximal M1 occlusion and medullary AVS (p=0.006), supporting the hypothesis that medullary AVS is significantly influenced by ischemic conditions in the territory of lenticulostriate arteries.

Conclusion

Three possible mechanisms for medullary AVS were considered: local ischemia in the DWM, ventriculopetal ischemic venous flow from the pial veins, and ventriculofugal ischemic venous flow from the basal ganglia. The results of the present study and the fact that the DWM is exclusively perfused by the cortical arteries favor the ventriculofugal flow hypothesis as the mechanism of medullary AVS. Although direct observation of the veins in the DWM by cerebral angiography is challenging, it can be deduced indirectly.

## Introduction

The deep white matter (DWM) contains transcerebral veins that directly connect the pial veins to the subependymal veins, as well as anastomotic medullary veins that connect the superficial medullary veins draining into the pial veins with the deep medullary veins draining into the subependymal veins [1,2]. Multiple studies using human and animal brains have reported the static anatomy of these veins [1-8]. However, direct and dynamic observation of these veins through current techniques, such as cerebral angiography, is still challenging due to spatial resolution issues.

Meanwhile, in acute ischemic stroke (AIS), asymmetrically prominent veins may appear in the affected hemisphere on blood-oxygen-level-dependent (BOLD) imaging, such as T2 star (T2*) magnetic resonance imaging (MRI) and susceptibility-weighted imaging (SWI). This phenomenon is referred to as the asymmetrical vein sign (AVS) and is observed in both the cortical venous system and the deep venous system. AVS reflects an increase in deoxyhemoglobin (DHb), a paramagnetic substance, resulting from elevated oxygen extraction fraction (OEF). When observed in the cortical veins, it has been reported to be associated with poor angiographic collateral flow to the occluded territory. AVS has also been identified as a marker of poor prognosis in AIS [9-12].

In cases of middle cerebral artery M1 occlusion, the cortex that suffered from ischemia experienced an increase in OEF, followed by a subsequent rise in DHb, a paramagnetic substance. This increase in DHb leads to the appearance of AVS in the cortical veins on T2* imaging. Additionally, the ischemia of the basal ganglia is caused by the occlusion of lenticulostriate arteries (LSAs), leading to AVS in the deep venous system.

However, concerning medullary AVS observed in DWM, it is necessary to understand its vascular anatomy, including the veins in DWM and the arteries perfusing DWM.

Arterial perfusion of the DWM is exclusively due to centripetal blood flow from the cortical artery, with no centrifugal blood flow [13]. As mentioned earlier, regarding the venous circulation of DWM, the superficial medullary veins drain into the pial vein, and the deep medullary veins drain into the deep venous system. Additionally, anastomotic medullary veins connect the superficial and deep medullary veins, and transcerebral veins connect the pial veins to the subependymal veins/deep venous system [1].

In this study, we tried to understand the dynamic venous flow in the DWM, which is difficult to observe directly by focusing on medullary AVS in patients with hyperacute cardioembolic occlusion of the horizontal segment of the middle cerebral artery (M1). This study includes exploring the underlying mechanisms leading to the appearance of medullary AVS, along with a detailed analysis of the hemodynamics of venous flow in the DWM in veins connecting the pial veins and deep venous system.

## Materials and methods

This retrospective cross-sectional study followed a protocol similar to the one used in our previous study on AVS in T2* MRI and angiographic collateral flow in acute M1 ischemic stroke patients [9], other than assessing AVSs on T2* images. The patient cohort analyzed in this study is identical to that used in our previous study [9].

Inclusion criteria

This study included AIS patients with M1 occlusion who underwent mechanical thrombectomy (MT) at Kameda Medical Center between July 2018 and December 2021.

Exclusion criteria

The exclusion criteria were as follows: patients with AIS who had tandem occlusions, patients who did not undergo T2*-weighted imaging, and patients with non-cardioembolic etiologies, such as intracranial atherosclerotic disease.

Protocol of cerebral angiography and mechanical thrombectomy

Hand injections of the target vessels are performed prior to treatment. Subsequently, the aspiration catheter and microcatheter are guided through the balloon-guiding catheter. MT is generally performed using a combined technique involving an aspiration catheter and a stent retriever.

Data collection

The patients underwent brain MRI, including diffusion-weighted imaging (DWI), fluid-attenuated inversion recovery, three-dimensional time-of-flight magnetic resonance angiography, and T2*-weighted imaging, using a 1.5 T MRI system (Avanto, Siemens Medical, Germany). The patients' backgrounds, such as underlying diseases, were also analyzed.

Definition of medullary AVS

**Figure 1:**
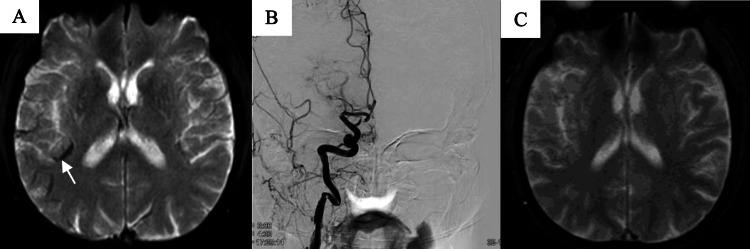
Sample T2* magnetic resonance images showing medullary asymmetrical vein sign A: T2* images of a case with right M1 proximal occlusion. Medullary AVS are visible on the affected side (white arrow). B: No LSAs are visible on the preoperative right ICA injection. C: Postoperative (within 48 hours) T2* images show no medullary AVS. LSAs: lenticulostriate arteries; ICA: internal carotid artery; AVS: asymmetrical vein sign

Medullary AVS on T2*-weighted imaging was defined based on our previous study [9] as the presence of more veins in the DWM on the affected side compared to the DWM on the intact side or veins within the DWM on the affected side being one and a half times wider than the diameter of the corresponding veins on the unaffected side. Figure 1 shows a case example featuring medullary AVS.

In this research, MRI images were evaluated separately by a skilled neurologist (Y.A.) and a seasoned neuroendovascular surgeon (K.K.). The neurologist (Y.A.) did not have access to patients' information. When any discrepancies arose, the two evaluators and an additional experienced neuroradiologist (Y.K.) deliberated the MRI findings until a consensus was achieved.

Evaluation of cerebral angiography

Cerebral angiography of the ICA on the affected side was independently evaluated by two seasoned neuroendovascular surgeons (T.M. and K.K.) in accordance with the trial design by the American Society of Interventional and Therapeutic Neuroradiology/Society of Interventional Radiology (ASITN/SIR) [14].

The participants were categorized into two groups based on the occlusion sites. The M1 proximal group included patients with occlusions of the M1 at or proximal to the LSAs, as indicated by cerebral angiography showing the absence of LSAs. The M1 distal group consisted of patients with occlusions of the M1 beyond the LSAs, including those with partial occlusion of the LSAs.

The collateral flow grade from the affected side of the anterior cerebral artery was assessed based on the trial design from the ASITN/SIR criteria [14]. Grade 0 was defined as no collaterals to the ischemic site were visible. Grade 1 indicated slow collaterals to the periphery of the ischemic site, with some persistence of the defect. Grade 2 referred to rapid collaterals to the periphery of the ischemic site, with the persistence of some defects and involvement of only a portion of the ischemic territory. Grade 3 represented collaterals with slow but complete angiographic blood flow to the ischemic bed in the late venous phase. Finally, Grade 4 denoted complete and rapid collateral blood flow to the vascular bed in the entire ischemic territory via retrograde perfusion.

In cases of differing opinions, the two evaluators, along with a seasoned neuroendovascular surgeon (M.T.), reviewed angiography up to the point where a consensus was achieved.

Grades 0, 1, and 2 were designated as poor collateral flow, while Grades 3 and 4 were classified as good collateral flow.

Data analysis

Demographic data were collected from patients, and the means and standard deviations for continuous variables were determined. For categorical variables, we calculated the counts and proportions. To evaluate differences between groups, Welch's t-test was used for continuous variables, while Fisher's exact test was employed for categorical variables. Statistical significance was set at p<0.05. All analyses were conducted using the R programming language (The R Foundation, Vienna, Austria) [15].

## Results

Participants

From July 2018 to December 2021, 100 AIS patients received MT. A total of 50 of these patients suffered from M1 occlusions. Among these, two patients had tandem lesions, and seven patients did not undergo T2* imaging and were therefore excluded from the study. This left a total of 41 patients for inclusion in the study (mean age was 78.4±10.5 years). Of these, 22 patients were classified into the poor collateral flow group, while 19 were categorized into the good collateral flow group.

Regarding the occlusion site, 19 patients had M1 proximal occlusion, and 22 had M1 distal occlusion. Nine patients had medullary AVS. None of the patients had developmental venous anomalies.

Association between angiographic findings and medullary AVS

In the good collateral flow group (n=19), four (21.1%) patients had medullary AVS, while in the poor collateral flow group (n=22), five (22.7%) were found to have medullary AVS. Univariate analysis revealed no significant relationship between collateral flow grade on angiography and the presence of medullary AVS (p=1.00).

With regard to the location of the occlusion, eight of 19 patients with M1 proximal occlusion had medullary AVS, while only one of 22 patients with M1 distal occlusion had medullary AVS. Thus, there was a significant association between M1 proximal occlusion and the presence of medullary AVS (p=0.006). The characteristics of patients with and without medullary AVS are shown in Table 1.

**Table 1:** Comparison of parameters between patients with and without medullary AVS Welch's t-test was used for continuous variables, and Fisher's exact test was used for categorical variables. AVS: asymmetrical vein sign; P2P: picture (MRI) to puncture; O2P: onset to picture (MRI); SD: standard deviation

Parameter	Medullary AVS (+) (N=9)	Medullary AVS (-) (N=32)	p-value
Age (years, mean±SD)	76.9±9.53	78.8±11.0	0.608
Sex, male	6 (66.7%)	17 (53.1%)	0.706
Sex, female	3 (33.3%)	15(46.9%)	0.706
Laterality, right	5 (55.6%)	17 (53.1%)	1.000
Laterality, left	4 (44.4%)	15 (46.9%)	1.000
Location, M1 proximal	8 (88.9%)	11 (34.4%)	0.006
Poor collateral grade	5 (55.6%)	17 (53.1%)	1.000
Good collateral grade	4 (44.4%)	15 (46.9%)	1.000
P2P time (min, mean±SD)	36.7±12.9	42.6±8.7	0.290
O2P time (min, mean±SD)	358.11±371.88	298.88±269.61	0.665
Hypertension	6 (66.7%)	26 (81.3%)	0.384
Dyslipidemia	3 (33.3%)	9 (28.1%)	1.000
Diabetes mellitus	1 (11.1%)	8 (25.0%)	0.654
Smoking	3 (33.3%)	8 (25.0%)	0.680

## Discussion

This study examined the medullary AVS on T2* and its association with angiographic findings, specifically angiographic collateral flow grade and occlusion location, in patients with hyperacute cardioembolic M1 occlusion. The analysis showed no significant association between medullary AVS and the angiographic collateral flow grade. However, a significant association was found between the medullary AVS and proximal M1 occlusion.

Based on these results and a literature review, we hypothesized that the following three mechanisms are responsible for the presence of medullary AVS.

Three possible mechanisms of medullary AVS

Medullary AVS indicates the presence of venous flow with an elevated DHb concentration in the DWM, which is derived from ischemia in the superficial and deep medullary veins. In addition, the transcerebral veins and anastomotic medullary veins connect the superficial and deep medullary veins [1,2].

Hence, we considered three possible mechanisms for the development of venous flow with elevated DHb concentration in the DWM.

The first mechanism involves a local elevation of OEF and DHb concentration due to the DWM ischemia. The second mechanism is an increase in OEF and DHb concentration in cortical veins as a result of cortical ischemia. In this scenario, venous flow in the transcerebral and anastomotic medullary veins is transported ventriculopetally through the DWM. Finally, the third mechanism involves increased OEF and DHb concentration in the deep venous system due to ischemia in the basal ganglia. In this case, venous flow from the transcerebral and anastomotic medullary veins is transported ventriculofugally through the DWM.

Angioarchitecture of the DWM

According to a study by Nonaka et al., blood flow to the DWM is fundamentally described as centripetal from the cortical arteries [13]. It has been suggested that no branches from the LSAs or choroidal arteries supply the DWM centrifugally [13].

While cortical arteries typically exhibit centripetal flow, there are exceptional cases with centrifugal flow, such as choroidal anastomosis in moyamoya disease or LSAs functioning as feeding arteries for arteriovenous malformations. However, the cases in this study did not fall into these special categories. Therefore, it is reasonable to consider that the DWM was primarily perfused by branches from the cortical artery in the patients included in this study.

Regarding the veins of the DWM, there are superficial medullary veins, which drain into pial veins as part of the superficial parenchymal veins, and deep medullary veins, which drain into the deep venous system. In addition to these veins with a fixed flow direction, anastomotic medullary veins connect these medullary veins, and transcerebral veins connect pial veins and the deep venous system/subependymal veins. There is no information in the literature regarding the flow direction of these veins. The transcerebral vein plays a role as a collateral pathway between pial and deep parenchymal veins and in the development of medullary veins [1].

Verifications of the three theories

Based on the discussion so far, several points are considered "already known." First, the DWM is perfused exclusively by cortical arteries. Second, there is an association between medullary AVS and the location of occlusion, specifically proximal M1 occlusion. Finally, there is no association between medullary AVS and leptomeningeal collateral flow.

If the first theory (increase in local OEF and DHb concentration due to ischemia in the DWM itself) is accurate, medullary AVS should be associated with leptomeningeal collateral flow because cortical arteries perfuse the DWM. However, this contradicts what is "already known."

If the second theory (elevated OEF and DHb concentration in pial veins due to cortical ischemia, with ventriculopetal venous flow in the DWM through transcerebral veins and anastomotic medullary veins) is true, the occurrence of medullary AVS should be directly influenced by the leptomeningeal collateral flow. Therefore, this theory also contradicts what is "already known."

Only the third theory (elevated OEF and DHb concentration in the deep venous system due to ischemia of the basal ganglia, with ventriculofugal venous flow in the DWM, such as transcerebral veins and anastomotic medullary veins) is compatible with what is "already known."

From these considerations, the mechanism of medullary AVS seems to involve veins connecting pial veins and deep venous systems, coursing through the DWM in a ventriculofugal direction. It is possible that medullary AVSs do not appear when these veins are not well-developed or when the flow direction is ventriculopetal.

However, all patients in this study had some ischemic symptoms due to M1 occlusion, indicating that they primarily had ischemia of the cortex and the DWM regardless of the leptomeningeal collateral flow. Therefore, we cannot conclude that this ventriculofugal venous flow is simply the sole and complete reason for the medullary AVS.

Clinical application

In clinical practice, performing MRI on patients prior to MT often results in motion artifacts that can distort the MRA, making it challenging to determine whether the occlusion site is in the proximal or distal M1 segment. Although not directly related to the direction of venous drainage in DWM, this study suggests that medullary AVS on T2* imaging can assist in determining whether the occlusion site is located in the proximal M1 segment. Being able to estimate the occlusion site before performing cerebral angiography can be beneficial for pre-selecting appropriate devices for the procedure.

Limitations

This study has two main limitations. First, T2*-weighted imaging was used as the BOLD imaging modality instead of SWI due to its shorter duration, allowing for quicker treatment initiation. Another reason for choosing T2* over SWI is that SWI is more susceptible to motion artifacts, which is particularly relevant since AIS patients often struggle to remain still during scans. However, compared to SWI, it is possible that the AVSs identified in this study were underestimated using T2*-weighted imaging.

Second, to save time, we focused solely on cerebral angiography of the occluded side. The collateral flow evaluated in this study was limited to the leptomeningeal anastomosis supplied from the ipsilateral anterior cerebral artery. We did not examine the internal carotid artery on the unaffected side or the vertebral arteries using cerebral angiography. As a result, the collateral flow may have been underestimated.

## Conclusions

Medullary AVS could be associated with ventriculofugal venous flow through transcerebral veins and anastomotic medullary veins in patients with hyperacute cardioembolic M1 occlusion. Since direct observation of venous flow in the DWM is difficult, BOLD imaging and an understanding of the vascular anatomy of the DWM might enable us to indirectly infer the status of blood flow in the white matter. Furthermore, in clinical practice, the presence of medullary AVS was found to suggest that the occlusion site is located in the proximal M1 segment.
